# Reduced cerebrospinal fluid concentration of interleukin-12/23 subunit p40 in patients with cognitive impairment

**DOI:** 10.1371/journal.pone.0176760

**Published:** 2017-05-02

**Authors:** Per Johansson, Erik G. Almqvist, Anders Wallin, Jan-Ove Johansson, Ulf Andreasson, Kaj Blennow, Henrik Zetterberg, Johan Svensson

**Affiliations:** 1 Department of Neuropsychiatry, Skaraborg Central Hospital, Falköping, Sweden; 2 Institute of Medicine, Sahlgrenska Academy, University of Gothenburg, Gothenburg, Sweden; 3 Department of Endocrinology, Skaraborg Central Hospital, Skövde, Sweden; 4 Department of Psychiatry and Neurochemistry, Institute of Neuroscience and Physiology, Sahlgrenska Academy, University of Gothenburg, Mölndal, Sweden; 5 UCL Institute of Neurology, Queen Square, London, United Kingdom; Nathan S Kline Institute, UNITED STATES

## Abstract

**Background:**

The role of inflammation in Alzheimer’s disease (AD) and other cognitive disorders is unclear. In a well-defined mono-center population, we measured cytokines and chemokines in paired serum and cerebrospinal fluid (CSF) samples.

**Methods:**

Consecutive patients with AD (n = 30), stable mild cognitive impairment (SMCI, n = 11), other dementias (n = 11), and healthy controls (n = 18) were included. None of the subjects was treated with glucocorticoids, cholinesterase inhibitors, or non-steroidal anti-inflammatory drugs. Serum and CSF concentrations of interleukin-6 (IL-6), IL-8, IL-12/23 p40, IL-15, IL-16, vascular endothelial growth factor-A (VEGF-A), and three chemokines were measured using a multiplex panel.

**Results:**

After correction for multiple comparisons, only CSF IL-12/23 p40 concentration differed significantly between the total patient group (n = 52) and controls (n = 18; p = 0.002). Further analyses showed that CSF IL-12/23 p40 concentration was decreased in all patient subgroups (AD, other dementias, and SMCI) compared to healthy controls (p < 0.01, p < 0.05, and p < 0.05, respectively). In the total study population (n = 70), CSF IL-12/23 p40 concentrations correlated positively with CSF concentrations of β-amyloid_1-42_ (Aβ_1–42_) and phosphorylated tau protein (P-tau) whereas in AD patients (n = 30), CSF IL-12/23 p40 only correlated positively with CSF P-Tau (r = 0.46, p = 0.01).

**Conclusions:**

Most cytokines and chemokines were similar in patients and controls, but CSF IL-12/23 subunit p40 concentration was decreased in patients with cognitive impairment, and correlated with markers of AD disease status. Further studies are needed to evaluate the role of CSF IL-12/23 p40 in other dementias and SMCI.

## Introduction

Cytokines are small proteins that can exert autocrine, paracrine, and endocrine actions [1]. Although cytokines may have specific functions, most functions can be stimulated also by other cytokines [1]. Some cytokines, like interleukin-6 (IL-6), are proinflammatory whereas other cytokines regulate the proinflammatory cytokine response [1]. The major function of chemokines is to induce migration of inflammatory cells [1].

In Alzheimer’s disease (AD), amyloid-β (Aβ) deposits and neurofibrillary tangles provide stimuli for inflammation, and activated immune cells and microglia are accumulated in the AD brain [2]. The neuroinflammation in AD might not only be a consequence of Aβ mismetabolism, but immune system-mediated actions could also drive AD pathogenesis [2]. Although overexpression of IL-6 or IL-1β produced inconclusive results in terms of amyloid load and tau accumulation in mouse AD models [3, 4], two recent studies in mice showed that IL-12 and IL-23 was upregulated in the senescent/AD brain, and experimentally induced inactivation of the common IL-12 and IL-23 subunit p40 reduced amyloid load and improved cognitive functions [5, 6]. Furthermore, administration of anti-inflammatory agents such as non-steroidal anti-inflammatory drugs (NSAIDs) reduced amyloid plaque load and microglial activation in animal models [7]. Human observational studies suggest that especially long-term use of anti-inflammatory drugs could reduce the risk of AD [8, 9], but randomized controlled trials have so far showed no clear effect on AD progression [10, 11].

In a meta-analysis of cytokines in AD, circulating (blood) concentrations of tumor necrosis factor (TNF)-α, transforming growth factor (TGF)-β, IL-1β, IL-6, IL-12 and IL-18 were increased in AD patients whereas in CSF, only TGF-β was elevated [12]. Dementing disorders other than AD have different underlying pathogenesis and clinical presentation, but changes in inflammatory markers and neuroinflammation can be observed also in other dementias [13, 14]. In another meta-analysis, higher peripheral concentrations of C-reactive protein (CRP) and IL-6 were associated with an increased risk of all-cause dementia whereas the association with risk of AD alone was weak [15]. Furthermore, although the mechanisms underlying neuroinflammation might differ depending on the cognitive disorder studied, some changes might also be overlapping. For instance, vascular pathology in the brain, which have been associated with inflammatory markers [15], can frequently be seen at postmortem examination in several neurodegenerative diseases [16].

Although inflammation is involved in cognitive decline, the nature of this involvement is still not fully clear. In a well-characterized mono-center cohort of patients with cognitive impairment and matched healthy controls, we determined serum and CSF concentrations of cytokines and chemokines using a multiplex panel. We also studied whether there were associations with CSF levels of AD biomarkers.

## Materials and methods

### Study participants

The study participants as well as AD CSF biomarkers have been reported previously [17]. The study consisted of consecutively recruited Caucasian patients admitted by their general practitioner for evaluation of cognitive impairment to a memory clinic in Falköping, Sweden. The participants were recruited by a single specialized physician (P.J.) 2000–2008. Inclusion criteria, beside being referred to Falköping Hospital for evaluation of suspected dementia, were age 65–80 years, body mass index (BMI) 20–26 kg/m^2^, and waist:hip ratio 0.65–0.90 in women and 0.70–0.95 in men. Exclusion criteria were serum creatinine > 175 mmol/L, diabetes mellitus, previous myocardial infarction, malignancy including brain tumor, subdural hematoma, ongoing alcohol abuse and previous or present medication with glucocorticoids or acetylcholine esterase inhibitors.

Control subjects were recruited contemporaneously from the same geographical area among spouses of the included patients and by advertisements in local newspapers. The controls had no subjective symptoms of cognitive dysfunction but otherwise, inclusion and exclusion criteria were similar as those in the patients. Totally, 60 patients and 20 healthy controls were recruited. However, in this analysis, patients treated with non-steroidal anti-inflammatory drugs (NSAIDs) were excluded. Therefore, 52 patients (29 men and 23 women) and 18 healthy controls (10 men and 8 women) were included in the present study.

The presence or absence of dementia was diagnosed according to the Diagnostic and Statistical Manual of Mental Disorders, Fourth Edition (DSM-IV) criteria. Patients with dementia were classified as suffering of Alzheimer’s disease (AD) [18], vascular dementia (VaD) according to the requirements by NINDS-AIREN [19] or the guidelines by Erkinjuntti et al. for the subcortical type of VaD [20], and dementia with Lewy bodies (DLB) as described previously [17].

All diagnoses were assessed by an independent specialized physician at a university hospital that was blinded to the results of CSF biomarkers, otherwise the specialist physician had access to all clinical data [17]. Mild cognitive impairment (MCI) was diagnosed in patients with cognitive impairment that did not fulfil the criteria for dementia [21]. Patients with MCI were followed at least annually for a median of 3 (range 1–7) years to evaluate whether they later developed dementia. During the follow-up visits, 11 patients remained stable in MCI (SMCI). Others progressed, during the follow-up period, to dementia and were diagnosed with AD (n = 6) or VaD (n = 3). MCI patients diagnosed with AD on follow-up visits did not differ in CSF concentrations of the AD biomarkers β-amyloid_1-42_ (Aβ_1–42_), total-tau (T-tau) or phosphorylated tau protein (P-tau) from patients with established AD at baseline (data not shown). Totally, the study population consisted of AD dementia or MCI diagnosed with AD dementia upon follow-up (n = 30), other dementias (n = 11), SMCI (n = 11), and healthy controls (n = 18). The distribution of diagnoses in the other dementia group were VaD or MCI diagnosed with VaD upon follow-up (n = 7) and DLB (n = 4).

### Ethical considerations

The study was approved by the ethical committee at University of Gothenburg (ethical approval number 496–99, T 452–05). The specialist physician (P.J.) gave written information of the study and explained the study protocol to the controls as well as to the patients and caretaker if available. All participants provided both oral and written informed consent. A next of kin, caretaker or guardian consented on behalf of patients if the capacity to consent was compromised. However, in all cases, the patient's own opinion was asked and considered, and the patient was recruited in the study only when he or she agreed with this. The ethical committee approved this informed consent procedure. The study was conducted according to the principles in the Declaration of Helsinki.

### Cognitive and physical examination

Before the test day, a mini-mental state examination (MMSE) [22] was performed. On the test day morning with the patients in the fasted state, before lumbar puncture was performed, body weight was measured to the nearest 0.1 kg, body height was measured barefoot to the nearest 0.01 m, and body mass index (BMI) was calculated as the weight in kilograms divided by the height in meters squared. Waist circumference and hip girth was measured as described previously [17].

### CSF Sampling

All CSF samples were collected by lumbar puncture in the L3/L4 or L4/L5 interspace at the standardized time point 8.30–9.00 am. The first 12 mL of CSF was collected in a polypropylene tube and immediately transported to the local laboratory for centrifugation at 2.000g at +4°C for 10 minutes. The supernatant was pipetted off, gently mixed to avoid possible gradient effects, and aliquoted in polypropylene tubes that were stored at -80°C pending biochemical analyses, without being thawed and re-frozen.

### Blood samples

Blood samples were drawn in the morning in the fasted state and serum was prepared by centrifugation after coagulation at room temperature for 15–30 min, aliquoted and stored in cryotubes at -80°C pending biochemical analyses, without being thawed and re-frozen.

### Biochemical procedures

All biochemical analyses were performed with the analyst blinded to the clinical diagnoses and other clinical information. All CSF analyses were performed at the Clinical Neurochemistry Laboratory in Mölndal, Sweden, by experienced laboratory technicians. All analyses were done at one occasion, using the same batch of reagents. CSF Aβ_1–42_ concentrations were determined using the INNOTEST® ELISA assay technology (Innogenetics, Ghent, Belgium) [23]. The axonal damage marker CSF T-tau and CSF concentrations of tau phosphorylated at threonine 181 (P-tau181) were measured using INNOTEST® ELISA assays [24, 25].

Cytokines and chemokines were measured using the V-PLEX Human Cytokine 30-Plex Panel Kit according to instructions from the manufacturer (Cat#: K15054G, Meso Scale Diagnostics, Rockville, Maryland). IL-6, IL-8, IL-12/23 p40, IL-15, IL-16, vascular endothelial growth factor-A (VEGF-A), interferon-γ-inducible protein-10 (IP-10), monocyte chemotactic protein-1 (MCP-1), and macrophage inflammatory protein-1β (MIP-1β) were present at concentrations within the dynamic range of the assay in both serum and CSF, whilst IL-1α, IL-1β, IL-2, IL-4, IL-5, IL-7, IL-10, IL-12p70, IL-13, IL-17, tumor necrosis factor (TNF)-α, TNF-β, granulocyte macrophage-colony stimulating factor (GM-CSF), interferon-γ (IFN-γ), eotaxin, eotaxin-3, MCP-4, macrophage derived chemoattractant (MDC), MIP-1α, and thymus and activation-regulated chemokine (TARC) were below the limit of detection in serum and/or CSF and are therefore not reported. In terms of IL-12/23 p40, the relative error for the back calculated concentrations of the calibrator curve, using data from the two runs needed to complete the CSF samples, were below 12% for all calibrator points. The range spanned by the calibrators was 0.69–2840 pg/mL, and all CSF IL-12/23 p40 samples had a higher signal than the lowest calibrator.

### Statistical analyses

The descriptive statistical results are given as the median (25th-75th percentile) if not otherwise stated. For continuous variables, between-group differences were assessed using the non-parametric Kruskal-Wallis test for comparisons between multiple groups, followed by the Mann-Whitney U test for pair-wise comparisons. Chi-square tests were used for group comparisons of categorical variables. Correlations were sought using the Spearman rank order correlation test. Significance was obtained if the two-tailed p-value was ≤ 0.05.

## Results

### Serum and CSF concentrations of cytokines and chemokines

Cytokine and chemokine concentrations in serum and CSF are given in Table 1. In serum as well as in CSF, the concentrations of the cytokines IL-6, IL-8 and IL-15, the cytokine/growth factor VEGF-A, and the chemokines IP-10 and MIP-1β were similar in the total group of patients (n = 52) compared to the controls (n = 18) (Table 1). In the patient group, serum concentrations of IL-16 and MCP-1 were decreased (p = 0.004 and p = 0.03 *vs*. controls, respectively) while CSF concentrations of IL-16 and MCP-1 were unchanged. However, the reductions in serum IL-16 and MCP-1 concentrations lost statistical significance after adjustment for multiple comparisons [18 cytokine levels were analyzed (9 in serum and 9 in CSF); Bonferroni correction: p-value of 0.05 divided by 18 (≤ 0.0028) was considered significant]. Serum IL-12/23 p40 was unaffected whereas CSF IL-12/23 p40 was decreased in patients compared to controls (p = 0.0020), and this difference remained statistically significant also after correction for multiple comparisons. Further analyses were therefore only performed in terms of IL-12/23 p40 levels.

**Table 1:** Serum and CSF levels of cytokines and chemokines (pg/mL) in 52 patients with cognitive impairment and 18 healthy matched controls.

	Patients (n = 52)	Controls (n = 18)	Between-group p-value
***Serum values***			
IL-6	0.88 (0.81–1.54)	1.04 (0.84–1.78)	0.19
IL-8	15.0 (10.8–18.9)	14.4 (11.6–19.2)	0.91
IL-12/23 p40	117 (79–157)	143 (102–185)	0.12
IL-15	2.00 (1.74–2.34)	1.91 (1.54–2.13)	0.23
IL-16	249 (195–328)	345 (289–482)	0.0044
VEGF-A	162 (103–246)	171 (109–254)	0.97
IP-10	252 (193–359)	293 (234–461)	0.23
MCP-1	310 (250–400)	412 (319–477)	0.03
MIP-1β	135 (108–158)	112 (85–184)	0.59
***CSF values***			
IL-6	1.14 (0.78–1.47)	1.18 (0.96–1.62)	0.29
IL-8	51.5 (41.0–64.4)	55.4 (43.9–66.9)	0.59
IL-12/23 p40	3.58 (3.01–4.60)	4.73 (4.08–6.29)	0.0020
IL-15	3.19 (2.76–3.92)	3.51 (3.04–4.12)	0.28
IL-16	7.59 (5.97–9.52)	8.50 (6.83–8.88)	0.35
VEGF-A	4.87 (4.17–5.48)	4.71 (4.56–5.01)	0.89
IP-10	539 (410–806)	631 (402–769)	0.97
MCP-1	514 (435–625)	450 (375–547)	0.11
MIP-1β	11.9 (10.4–13.6)	10.3 (9.2–13.5)	0.32

The unit is pg/mL for all variables. Values are given as the median (25th-75th percentile). P-values in the right column were calculated using the Mann-Whitney U test.

### CSF IL-12/23 p40 according to clinical diagnosis

In the additional analyses, the patient group was divided according to the clinical diagnoses (Table 2). The patients and healthy controls were comparable in terms of age, gender, BMI, and waist: hip ratio. AD biomarkers in CSF have been reported previously [17]. None of the investigated variables in serum or CSF correlated with age or CSF/serum albumin ratio (data not shown).

**Table 2:** Anthropometric measures, MMSE score, and CSF AD biomarkers in 52 patients with cognitive impairment and 18 healthy matched controls.

	AD (n = 30)	Other dementias (n = 11)	SMCI (n = 11)	Controls (n = 18)	P-value
**Men/women**	15/15	9/2	5/6	10/8	0.27
**Age (years)**	74 (71–77)	74 (72–76)	72 (69–73)	76 (71–78)	0.26
**BMI (kg/m**^**2**^**)**	22.8 (21.7–25.1)	22.8 (20.4–25.3)	24.1 (23.2–26.2)	23.7 (22.7–25.2)	0.22
**Waist/hip ratio**	0.86 (0.78–0.91)	0.90 (0.88–0.93)	0.85 (0.79–0.90)	0.87 (0.83–0.91)	0.17
**MMSE score**	23 (19–25)^**a**^^,^^**b**^	22 (19–24)^**b**^^,^^**c**^	28 (27–29)	29 (27–29)	< 0.0001
**CSF Aβ**_**1–42**_ **(pg/mL)**	409 (329–486)^**a**^^,^^**b**^	390 (337–507)^**c**^^,^^**d**^	671 (548–883)^**e**^	990 (786–1044)	< 0.0001
**CSF T-tau (pg/mL)**	584 (413–782)^**a**^^,^^**b**^^,^^**f**^	311 (254–374)	270 (211–398)	327 (224–390)	< 0.0001
**CSF P-tau (pg/mL)**	104 (78–116)^**b**^^,^^**c**^^,^^**f**^	46 (34–61)^**e**^	60 (37–79)	66 (56–80)	< 0.0001

Values are given as the median (25th-75th percentile). The *P*-values in the right column refers to differences between all four groups using the Kruskal-Wallis test. Comparisons between two separate groups were performed using the Mann-Whitney U test.

^**a**^ p < 0.0001 *vs*. controls

^**b**^ p < 0.001 *vs*. SMCI

^**c**^ p < 0.001 *vs*. controls

^**d**^ p < 0.05 *vs*. SMCI

^**e**^ p < 0.05 *vs*. controls

^**f**^ p < 0.0001 *vs*. other dementias.

**Fig 1:**
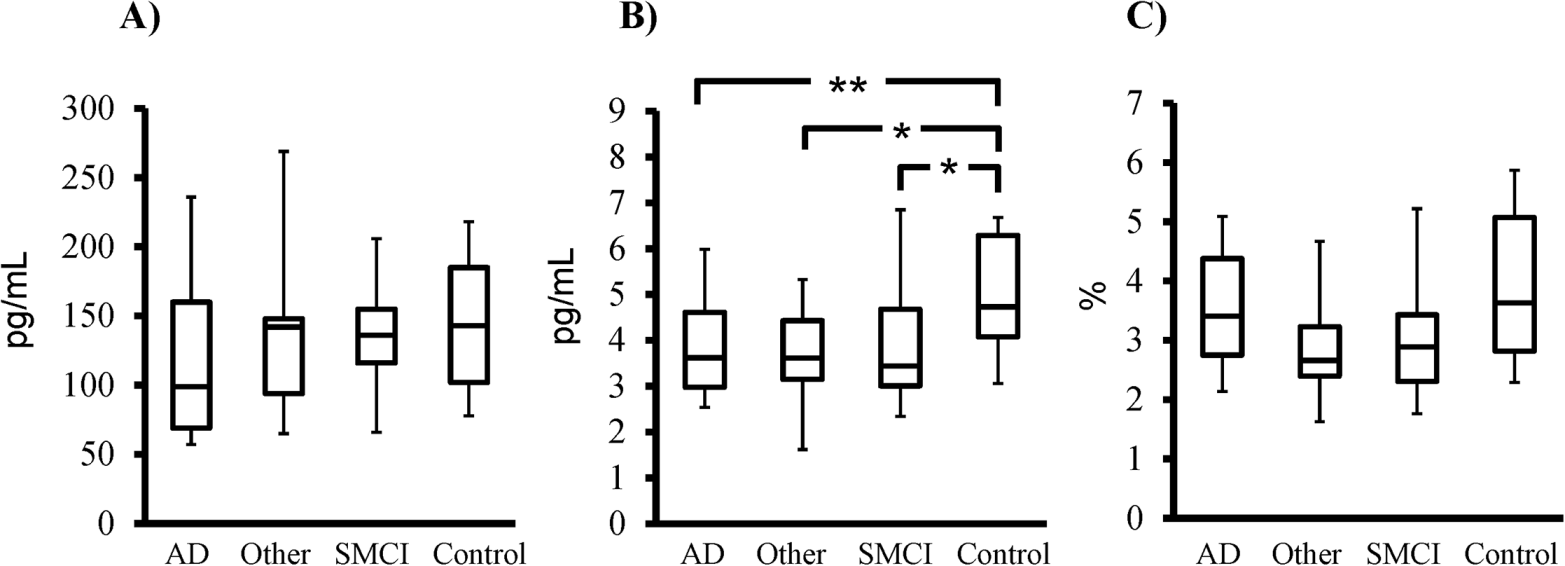
Reduced CSF IL-12/23 p40 concentration in patients with cognitive impairment. (A) Serum IL-12/23 p40 concentration, (B) CSF IL-12/23 p40 concentration, and (C) CSF/serum IL-12/23 p40 ratio in the study population of AD patients (n = 30), other dementias (n = 11), SMCI (n = 11), and healthy controls (n = 18). Values in the box plots are given as medians (horizontal lines), 25th-75th percentiles (boxes), and 10th-90th percentiles (whiskers). Differences between multiple groups were assessed using the Kruskal-Wallis test, followed by the Mann-Whitney U test for pair-wise comparisons. * p < 0.05, ** p < 0.01 vs. control group.

Serum IL-12/23 p40 did not differ between the study groups (Fig 1A) whereas CSF IL-12/23 p40 was decreased in all patient subgroups (AD, other dementia, and SMCI) compared to the controls (p < 0.01, p < 0.05, and p < 0.05, respectively; Fig 1B). The ratio between CSF and serum values of IL-12/23 p40 did not differ between groups (Fig 1C).

### Correlation analysis

**Fig 2:**
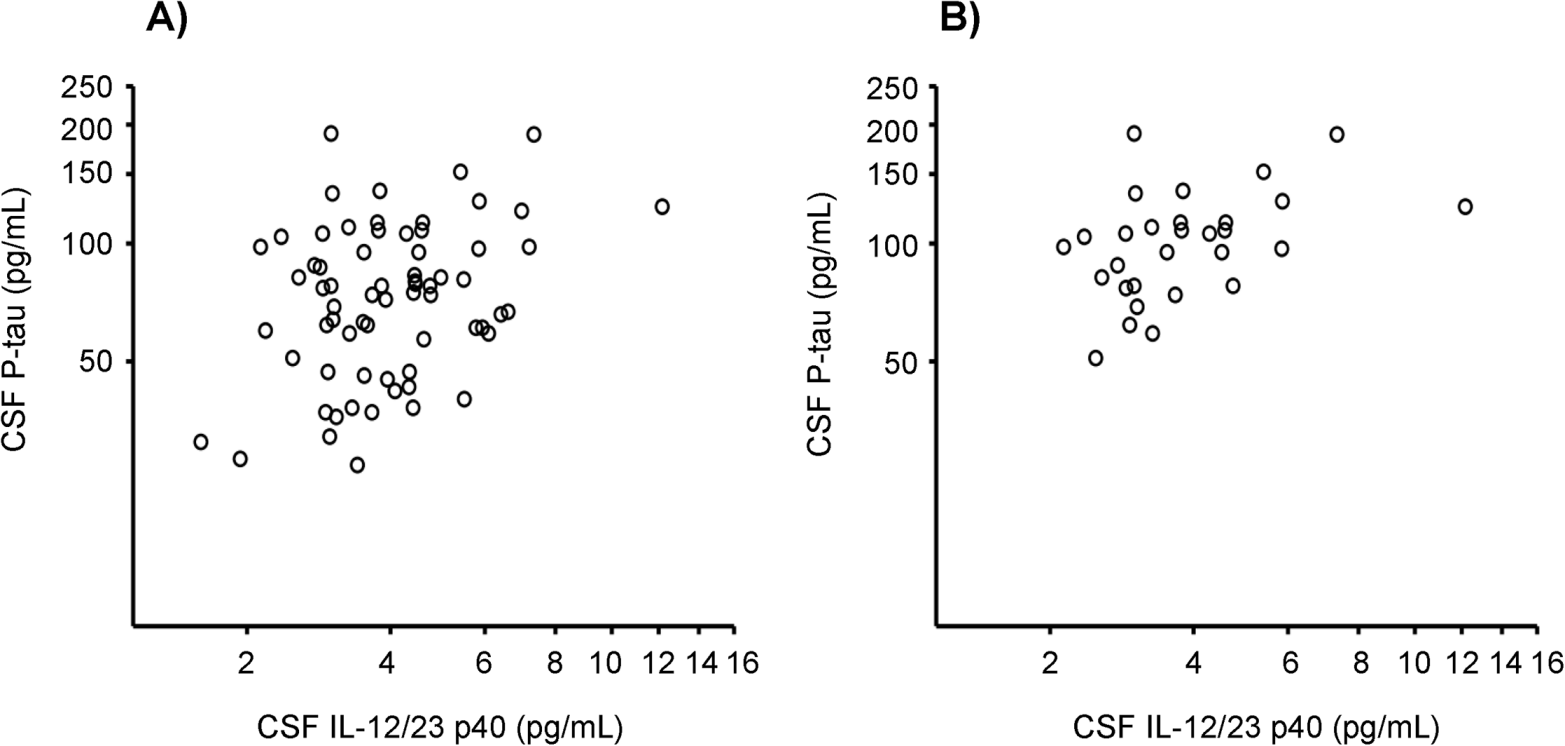
CSF IL-12/23 p40 correlates positively with the CSF AD biomarker phosphorylated tau protein (P-tau). (A) In the total study population (n = 70; r = 0.25, p < 0.05) as well as (B) in the AD group (n = 30; r = 0.46, p = 0.01), CSF IL-12/23 p40 concentration correlated positively with CSF P-tau concentration. Note the logarithmic scale on both the x-axis and the y-axis. Correlations were sought using the Spearman rank order correlation test.

We evaluated whether CSF IL-12/23 p40 correlated with CSF AD biomarkers and MMSE score. In the total study population (n = 70), CSF IL-12/23 p40 concentrations correlated positively with CSF concentrations of Aβ_1–42_ (r = 0.24, p < 0.05) and P-tau (r = 0.25, p < 0.05; Fig 2A) but not with CSF T-tau (r = 0.06) or MMSE score (r = 0.04). In AD patients (n = 30), CSF IL-12/23 p40 correlated positively with CSF P-tau (r = 0.46, p = 0.01; Fig 2B) but not with Aβ_1–42_ (r = 0.04), T-tau (r = 0.27), or MMSE score (r = -0.23).

## Discussion

In this mono-center study, strictly defined procedures were followed in terms of clinical assessments and diagnostic procedures including lumbar puncture [17]. The patients and controls were matched in terms of age, gender, BMI, and waist: hip ratio [17]. None of the participants had diabetes mellitus or received medical treatment with glucocorticoids, NSAIDs, or acetylcholine esterase inhibitors. The latter medication may be a confounder in studies of AD as acetylcholine may modulate inflammatory responses [26]. Thus, several factors that could influence cytokine concentrations were highly standardized. A limitation of the present study is the cross-sectional design, and changes over time could therefore not be studied.

After corrections for multiple comparisons, serum as well as CSF concentrations of the cytokines IL-6, IL-8, IL-15, IL-16, and the cytokine/growth factor VEGF-A were not significantly altered in the total patient group (n = 52) compared to the control group (n = 18). Several studies including a meta-analysis have shown increased serum IL-6 concentration in AD patients [12, 27, 28], whereas most [12, 29] but not all [30] studies have shown unchanged CSF IL-6 concentration in AD or VaD. Moreover, circulating or CSF concentrations of IL-8, IL-15, IL-16, and VEGF have been decreased [31, 32], unchanged [12, 33–35], or increased [34, 36–39] in AD. These relatively variable results in AD and VaD could, at least partly, be explained by the fact that quantification of low cytokine levels in CSF is technically difficult. However, we used highly standardized procedures to reduce measurements errors. Serum and CSF samples had previously not been thawed, and the analyses were performed by experienced laboratory technicians blinded to all clinical information.

In line with the results of other studies, we observed that concentrations of the chemokines IP-10 and MCP-1 were higher in CSF than in serum in both controls and patients [37, 40]. However, serum as well as CSF concentrations of IP-10, MIP-1β, and MCP-1 were similar in the total patient group compared to the control group after adjustment for multiple comparisons. In previous studies, unchanged [40, 41] or increased [37, 42] levels of IP-10 and MCP-1 have been found in blood or CSF of AD patients. Immunohistochemistry revealed elevated IP-10 that co-localized with MIP-1β in astrocytes in AD brains, and IP-10 positive astrocytes were associated with senile plaques [43]. Therefore, the expression of some chemokines might be upregulated in astrocytes in the AD brain, but our results do not suggest that this is associated with any major changes in their CSF concentrations.

CSF IL12/23 p40 concentration was reduced in the total patient group (n = 52) compared to the control group (n = 18), and this difference remained also after correction for multiple comparisons. IL-12 is a heterodimeric cytokine formed by a 35 kDa light chain (the p35 subunit encoded by *IL12A*) and a 40 kDa heavy chain (the p40 subunit encoded by *IL12B*) [44]. The p40 subunit of IL-12 is shared with IL-23 [44, 45]. Both IL-12 and IL-23 has been implicated in bacterial infections and autoimmune diseases [44, 45]. Furthermore, single nucleotide polymorphisms (SNPs) in *IL-12A* (rs2243115 and rs568408) [46], *IL-12B* (rs3212227) [46], and IL-23 receptor gene (rs10889677 and rs1884444) [47] were associated with the risk of AD in a Chinese population, suggesting that the genes encoding IL-12 and IL-23 receptor could be risk factors for late-onset AD susceptibility [46, 47].

We divided the total patient group according to the clinical diagnoses, and observed that CSF IL12/23 p40 concentration was reduced in AD, other dementia, as well as SMCI compared to the healthy controls. As the number of patients with other dementia or SMCI was relatively low, we focused on the AD group (n = 30). A previous study in AD patients showed unchanged serum and CSF concentrations of IL-12/23 p40 [48], and CSF IL-12 concentration was unchanged [49] or reduced [50] in AD. However, our study included AD patients in relatively early phases of the disease (median MMSE score = 23), whereas the previous studies were performed in more advanced AD (mean MMSE scores between 16 and 18) [48–50].

In AD, MMSE score correlated negatively with CSF IL-12/23 p40 in one study [5], and CSF IL-12 correlated positively with CSF tau levels in another study [50]. We did not observe any correlation between CSF IL-12/23 p40 and MMSE score, but CSF IL-12/23 p40 correlated positively with CSF Aβ_1–42_ in the total study population (n = 70), and with CSF P-tau both in the total population (n = 70) and in AD patients (n = 30). These findings might suggest that there is an association between CSF IL-12/23 p40 concentration and markers of AD disease status. However, although CSF IL-12/23 p40 was reduced and CSF P-tau was increased in AD patients, the correlation between these analytes was positive. Therefore, the physiological importance of this correlation is not fully clear and needs to be explored in further studies.

In a previous study, CSF concentrations of Aβ_1–42_ as well as the pro-inflammatory protein S100A9 (MRP14) were decreased in AD patients and in the post-mortem brain, immunohistochemical analyses showed intense co-localized immunostaining of S100A9 and Aβ [51]. This possibly suggests that S100A9, like Aβ_1–42_, could be low in CSF of AD patients due to increased aggregation/sequestration of S100A9 in the AD brain. Furthermore, the production of IL-12/23 p40 by microglia was increased in the APPPS1 mouse model of AD, and peripheral administration of a neutralizing p40-specific antibody reduced cerebral amyloid load [5]. Moreover, using the senescence-accelerated mouse prone-8 (SAMP8) model, the expression of IL-12 and IL-23 in the brain were upregulated during aging, and knock down of the p40 subunit by *in vivo* infusion of interfering RNA decreased cerebral Aβ levels, reduced neuronal loss, and reversed cognitive impairment [6]. IL-12/23 p40 has therefore been shown to be of pathophysiological importance in animal studies of AD and it could be speculated that IL-12/23 p40, like Aβ_1–42_ and S100A9, could be low in CSF due to increased aggregation/sequestration of IL-12/23 p40 around AD plaques.

CSF IL-12/23 p40 was low also in patients with other dementias and SMCI. Few previous studies have analyzed IL-12/23 p40 or IL-12 concentrations in dementias other than AD. Serum and CSF IL-12/23 p40 were unchanged in VaD [48]. Nonamnestic multiple domain MCI was associated with higher serum IL-12 concentration [52], whereas patients with frontotemporal dementia had reduced CSF IL-12 compared to patients with non-inflammatory neurological diseases [50]. In our study, there were relatively few SMCI patients and the number of each specific diagnosis was low in the other dementia group. Therefore, the role of IL-12/23 p40 in cognitive disorders other than AD needs to be explored in further studies.

## Conclusions

In a homogenous, well-controlled study cohort, most of the cytokines and chemokines studied were similar in patients and controls after correction for multiple comparisons. CSF IL-12/23 p40 concentration was decreased in AD patients and correlated with markers of AD disease status. Based on previous animal data, it could be speculated that the reduced CSF IL-12/23 p40 concentration is secondary to aggregation/sequestration of IL-12/23 p40 around AD plaques. Furthermore, CSF IL-12/23 p40 was decreased also in other dementias or SMCI, but the number of patients in these groups was relatively small. Therefore, further studies are needed to clarify the role of CSF IL-12/23 p40 in dementing disorders other than AD.
